# Self-formation of compositionally complex surface oxides on high entropy alloys observed by accelerated atom probe tomography: a route to sustainable catalysts†

**DOI:** 10.1039/d4mh00245h

**Published:** 2024-07-18

**Authors:** Valerie Strotkötter, Yujiao Li, Aleksander Kostka, Florian Lourens, Tobias Löffler, Wolfgang Schuhmann, Alfred Ludwig

**Affiliations:** a Materials Discovery and Interfaces (MDI) Institute for Materials, Ruhr University Bochum Universitätsstraße 150 D-44801 Bochum Germany alfred.ludwig@ruhr-uni-bochum.de; b Center for Interface-Dominated High Performance Materials (ZGH) Ruhr University Bochum Universitätsstraße 150 D-44801 Bochum Germany; c Research Center Future Energy Materials and Systems (RC FEMS), Ruhr University Bochum Universitätsstraße 150 D-44801 Bochum Germany; d Analytical Chemistry – Center for Electrochemical Sciences (CES) Faculty of Chemistry and Biochemistry, Ruhr University Bochum Universitätsstraße 150 D-44801 Bochum Germany

## Abstract

Sustainable catalysts rely on abundant elements which are prone to oxidation. A route to non-noble electrocatalysts is opened by directing the formation of unavoidable surface oxides towards creating a few atomic layers of an active and stable electrocatalyst, which is in direct contact with its metallic, conducting support. This is enabled by combining possibilities of compositionally complex solid solutions with accelerated atomic-scale surface characterization. Surface composition changes from the as-synthesized state to states after exposure to the oxygen evolution reaction (OER) are investigated using a Cantor-alloy-catalyst-coated tip array for atom probe tomography (APT): The film on top of the tip forms a nanoreactor which enables acquisition of intrinsic properties. The as-deposited film has an around 3 nm thick native oxide; short and prolonged OER exposures result in an oxygen-influenced surface layer with lower oxidation depth and altered metal composition. This shows that as-synthesized complex compositions can be used to obtain active and stable surface oxides under electrochemical load, while their surface evolution is observed by accelerated APT.

## Abstract 2

Accelerated approach for analysis of electrochemical states with atom probe tomography reveals modification of oxygen-containing surface layers of high entropy catalysts.

## Graphical abstract

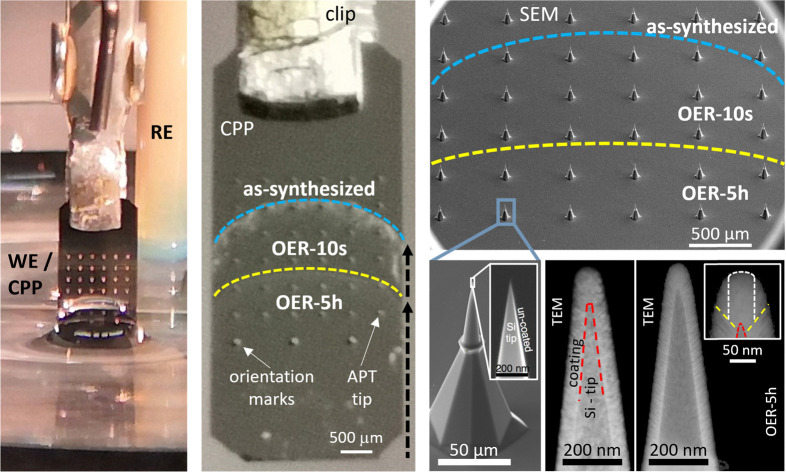

**New concepts**

Compositionally complex surfaces offer new design principles for electrocatalysts. However, this requires understanding of their surface composition down to the atomic level. This is already difficult for a single sample but becomes even more challenging when different samples in different states need to be compared. To address this, we present an electrochemical combinatorial processing platform approach which allows to perform all experiments on the same material using an array of 36 thin-film-coated tips ready for atom probe tomography. This allows accelerated characterization of different sample states: as-synthesized and after application of different electrochemical loads. Using this approach on the example of the Cantor alloy in the oxygen evolution reaction, we investigated the surface composition evolution by monitoring the redistribution of atoms upon exposure to electrochemical load and elucidated the self-formation of nanoscale surface oxides.

## Introduction

High entropy alloys (HEAs) or, more specifically, compositionally complex solid solutions (CCSS) consist of at least 5 different elements which are randomly distributed on the lattice sites of a typical HEA crystal structure such as face-centered cubic (fcc).^1^ CCSS exhibit unique features for catalysis as they provide a statistical distribution of different surface atom arrangements, which can act as active sites. The Cantor alloy – consisting of non-noble and mostly abundant elements – was one of the first HEA catalysts discovered.^2,3^ In CCSS many different active sites exist due to the different elemental environments, as the binding energy of an active site to the reactants is also determined by the interaction with neighboring atoms.^4,5^ This fundamental difference from other classes of catalysts is the basis for many of their special properties.^6,7^ However, to exploit the new possibilities of HEA catalysts it is necessary to determine the surface composition of CCSS with highest resolution, *i.e.* approaching the atomic scale and in dependence of the state of the catalyst in the as-synthesized conditions and after exposures to the ambient and to different electrochemical loads (type of electrochemical reaction, reaction parameters, time of exposure).

However, since sustainable CCSS catalysts consist of oxidation-prone metals, the catalytic surface will almost never be metallic. Instead, it will transform to an oxide or a mixture of oxides and residual metal atoms.^8^ Exceptions might occur in reductive processes with absence of oxygen in the electrolyte. This means that the real surface of the oxidation-prone CCSS will be different to the intended nominal composition.

Since oxidation under ambient conditions is unavoidable, it is of great importance to turn this “problem” to an advantage, *i.e.*, achieving that the metallic surface forms an active and stable oxide layer on the very surface of the material, while the volume of the material remains metallic and therefore maintains good electrical conductivity, unlike the mostly insulating oxides. Since the search space is complex due to the multidimensionality of HEAs, there is a need for a suitable method to efficiently identify the right materials, *i.e.*, materials which form protective oxides which act as active and stable catalysts. To address this challenge an accelerated near-atomic-scale investigation of complex surfaces is needed. For this, we introduce a novel high-throughput method based on combinatorial sputtering combined with atom probe tomography (APT) and electrochemistry: the electrochemical combinatorial processing platform (EC-CPP) approach. In this accelerated approach, a Si-microtip array is coated uniformly with the material of interest, resulting in a so-called combinatorial processing platform (CPP) with 36 identical atomic-scale-mixed thin films. CPPs were already successfully applied for the investigation of the phase evolution of CCSS on the example of the Cantor alloy (Cr_*x*_Mn_*x*_Fe_*x*_Co_*x*_Ni_*x*_) at different annealing conditions and thermal oxidation but not yet for electrochemical experiments.^9–11^ An advantage of the CPP approach is that on top of the tip, a thin film nanoreactor is formed. The film nanostructure on the top of the tip (typically larger grains) is usually different from the structure of the films which grow on the shank part of the tip (typically nanostructured, with very small grains and many grain boundaries).^12–14^ The film grown on the shank part can be used for studies of the influence of microstructure on film properties. However, the tip part can be used to determine intrinsic properties of the film, due to the nanoscale dimension of the film on top of the tip and its morphology: for electrochemistry this morphology is favorable as no transport limitations occur. Therefore, this first EC-CPP study focuses on the intrinisic properties of the nanoreactor film.

APT provides three-dimensional positions of individual atoms and their chemical identities at the near-atomic scale with high mass resolution,^15^ and thus is an excellent tool to analyze the elemental composition of a catalytic surface.^16,17^ Applications of APT for catalysis^18–22^ have elucidated the surface chemical composition following catalytic reactions, enhancing understanding of the composition–activity relationships of catalysts. For example, Xiang *et al.* demonstrated the use of APT for catalyst investigation before and after OER exposure:^23^ They elucidated the 3D structure of 10 nm Co_2_FeO_4_ and CoFe_2_O_4_ nanoparticles. The 3D compositional distribution of the catalyst material provides an atomic-scale view of the surface element composition and active sites. Nevertheless, since APT necessitates needle-shaped samples, as pointed out in a review,^16^ these APT experiments involve usually additional sample preparation and the need for surface protection, which can present practical challenges in the development of new materials such as sequential and time-consuming sample preparation with high likeliness of unwanted changes to the sample by the preparation.

As a first example of the EC-CPP approach, which requires neither sample preparation nor a surface protection layer, we investigate the surface of a near equiatomic Cantor alloy thin film electrocatalyst material to evaluate possible changes of the catalyst material after the oxygen evolution reaction (OER) in dependence of the exposure time to OER at a constant potential applied to the CPP. Due to the harsh oxidation conditions during the OER all catalysts form at least a surface oxide layer or completely dissolve.^24–26^ Therefore, significant changes in the catalyst surface can be expected, which are revealed using our new approach.

## Results and discussion

### The electrochemical combinatorial processing platform approach

**Fig. 1:**
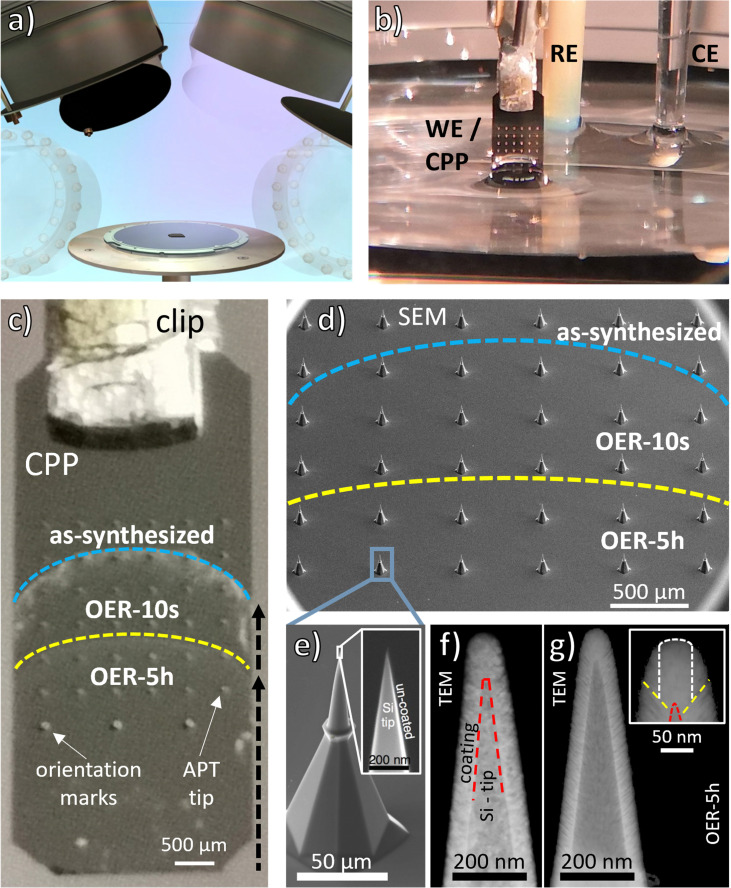
The EC-CPP approach. (a) Schematic of the deposition process applied on a Si tip array to form a CPP: one sputter cathode deposits an atomically mixed film from a CrMnNiCoFe alloy target with rotating substrate resulting in an approximately 100 nm thick homogeneous film. (b) Experimental three-electrode setup with an EC-CPP as working electrode (WE). (c) Image of an EC-CPP with 36 APT tips, different immersion depths 1 (up to yellow line) and 2 (up to blue line). (d) SEM image of a CPP, lines and resulting states as in 1c. (e) Magnified SEM image of an uncoated tip and its sharp end, which serves as substrate for the deposition of the thin film. (f) and (g) HAADF TEM images of CrMnNiCoFe coated and annealed tips, before and after the electrochemical exposure, respectively. The close up of the state after OER-5h is shown with yellow-dashed lines to indicate the estimated boundaries of the nanoreactor. White-dashed lines indicate the region of the performed APT analyses with a diameter of approx. 50 nm. Fig. 1(a), (d)–(f) adapted from Li *et al.*,^9^ copyright The Royal Society of Chemistry 2018.

The EC-CPP is realized by coating a pre-sharpened Si tip array uniformly with the catalyst material of interest, here a Cantor alloy thin film, with a thickness of about 100 nm using sputter deposition (Fig. 1(a)) and is then used as the working electrode (WE) for the OER. Fig. 1(b) shows the experimental setup. By controlling the number of tip-rows that are wetted by the electrolyte and the wetting time, different states of the EC-CPP tips are established in a single experiment. The immersion depth and time are controlled by a step motor-actuated micrometer screw. Due to the meniscus of the electrolyte, the EC-CPP shows a curved electrolyte surface line. The OER potential was applied at two different immersion depths for two different periods of time (Fig. 1(c)). By controling the tip depth, the bottom rows were exposed to the applied potential the longest time and the upper rows for the shortest. The top row remained unwetted for the analysis of the as-synthesized state. Fig. 1(d) shows the Si tip array on the EC-CPP in detail. As a proof-of-principle experiment for the new approach, an OER potential of 1.7 V *vs.* RHE was applied for a short (10 s) and a long (5 h) period of time to the EC-CPP. The potential of 1.7 V was chosen by analysing a linear sweep voltammogram (LSV, Fig. S1, ESI†) of a Cantor alloy thin film: at this potential the film is clearly in the OER range and simultaneously excessive bubble formation is avoided. Previous work on Cantor alloy oxide has also shown that 1.7 V is an appropriate value for the OER analysis.^27^Fig. 1(f) shows HAADF TEM images of an as-synthesized CPP tip and Fig. 1(g) another one after electrochemical exposure (OER-5h). The TEM images were taken with different TEMs leading to different contrast and resolution. The comparison between the images shows that the conformal coating is still observed after OER, *i.e.* no severe or even observable dissolution has taken place. Also, ICP-MS analysis of the electrolyte after 5 h of applied OER potential on a Cantor alloy thin film, synthesized with the same parameters, does not detect any elements above the detection limit. The nanoreactor (top of the film, shown in the inset with yellow-dashed lines), which is investigated with APT (measurement region indicated with white-dashed lines) is similar for both states and its intended film thickness was about 100 nm. The APT-observed reaction front is only a few nanometers thick and cannot be seen in these TEM images.

### APT analysis

**Fig. 2:**
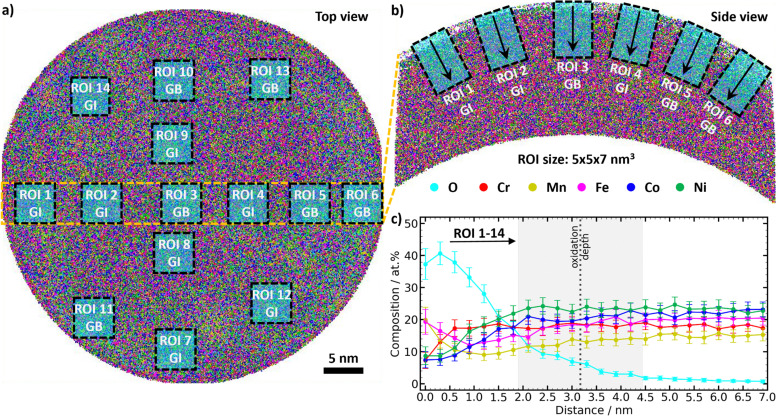
Results of the APT analysis of the as-synthesized state, measured on a representative tip from the EC-CPP. (a) Top view on the visualized APT data of the coated tip, and (b) side view of the 5-nm thin slice marked in yellow in the top view. The ROIs are marked by blue rectangles. The arrows show the direction of the composition profiles. (c) Depth-dependent composition plot of merged and averaged 14 ROIs from this tip. The mean oxidation depth (dashed line) and its standard deviation (grey area), as well as the compositional error of the APT measurements (error bars) are indicated.

The tips of the EC-CPP in their different states were investigated by APT. Fig. 2(a) and (b) show color-coded visualizations of the resulting data as a 3D tip reconstruction with depth-dependent element contents for a representative tip of the as-synthesized state. Similar images of tips from the states OER-10s and OER-5h are shown in ESI,† Fig. S2 and S3a, b, respectively. A first important finding is that the electrocatalytic film on the CPP withstands the extremely harsh exposure to the OER and can be measured by APT. For the analysis, evenly distributed regions of interest (ROIs) were taken from the different tips (Fig. 2(a) and (b)) to derive composition *vs.* depth plots, see Fig. S4 (ESI†). Depending on the position of the ROI in the grain, it was distinguished between grain interior (GI) and grain boundary (GB). In the Cantor alloy, GBs can be easily identified due to segregation of Mn and Ni at the GBs.^9,11,28^ In alloys without significant GB segregation, the different number densities of atoms at GBs compared to those in GIs, which are caused by trajectory aberrations of ions during their evaporation under high electric field in the APT,^29,30^ can be used to distinguish GBs from GIs.^31^ To ensure a good comparability, the ROIs were taken from 7 GIs and 7 GBs to achieve 14 ROIs in total for each tip. The oxygen-influenced surface layer is defined as the part of the film surface that contains a non-negligible oxygen content (O > 3 at%). This point is defined as “oxidation depth”. The oxidation depth is higher for the GB ROIs compared to the GI ROIs (ESI,† Fig. S2, S3, S5d and e, respectively). This is because GBs often act as preferred pathways for the diffusion of atoms. The irregular atomic structure in these regions enables atoms, including oxygen, to diffuse faster than in the GIs. This faster diffusion at GBs leads to quicker and deeper oxidation, see below.

To improve the statistical analysis, a consolidated data set was created by combining the data from the ROIs of a single tip (Fig. 2(c) and Fig. S2c, S3c, S5c, ESI†). The error of the oxidation depth results from the standard deviation of the oxidation depths of the different ROIs. For the error of the oxidation depth of the different states, the standard deviation from the oxidation depths of the different tips was taken. In order to obtain the data for the different states as-synthesized, OER-10s, and OER-5h, the whole procedure was repeated by combining the data from the tips of the respective states (ESI,† Fig. S6 and S7). These plots were used to analyze the oxidation depth and depth-dependent composition change in the oxygen-influenced surface layer. An overview about the data from the different tips is given in ESI,† Table S1.

### Oxidation behavior

The analysis of the oxidation depth in the as-synthesized state and under OER conditions shows that the oxygen-influenced surface layer exists already in the as-synthesized state as the oxophilic constituents form an oxidized surface as soon as the films get exposed to the ambient. Such native oxide layers are expected for oxophilic materials and were also observed for the Cantor alloy^32–34^ and other HEAs.^35–39^ In many studies, X-ray photoelectron spectroscopy (XPS) is used to investigate the chemical composition and bonding states at the surface. However, XPS is limited by its depth resolution. Sampling depths smaller than 5–10 nm (regular XPS) can only be achieved by angle-resolved XPS, while greater depths can be sampled by XPS depth profiling. In both cases, the depth resolution is inferior to that of APT. In addition, the ion etching process used in XPS depth profiling has the potential to unintentionally alter the samples’ oxidation state. The lateral resolution of XPS is on the scale of tens of microns, and therefore much less accurate than APT and unable to precisely measure the nanoreactor films. To obtain information about the chemical state of the Cantor alloy metals by XPS, peak fitting is necessary. However, in the case of Mn, Fe, Co, and Ni, this presents a significant challenge, as their main XPS peaks overlap with potential Auger peaks (Mn 2p – Ni LMM_a_, Fe 2p – Co LMM/Ni LMM_b_, Co 2p – Fe LMM_a_, Ni 2p – Mn LMM_c_). Nevertheless, XPS measurements were performed on an additional Cantor alloy film that was deposited on a flat substrate and measured before and after exposure to OER-5h (see Fig. S8 and S9, ESI†). The high-resolution XPS spectra of the as-synthesized film indicate the presence of both metallic and oxidized metal species. After OER-5h, the XPS spectra indicate fewer metallic and more oxidized species. This might be related to the nanostructure of the film grown on a flat substrate instead of the EC-CPP tips. TEM images of the nanoreactor do not show any visible grain boundaries (Fig. 1(g)), whereas films on flat surfaces (as well as on the shank part of the CPP tips, Fig. 1(g)) typically contain many grain boundaries, which will result in a different oxidation behavior. We focus on APT data, as only these provide three-dimensional compositional information of the intrinsic properties of the nanoreactor with the highest resolution.

**Fig. 3:**
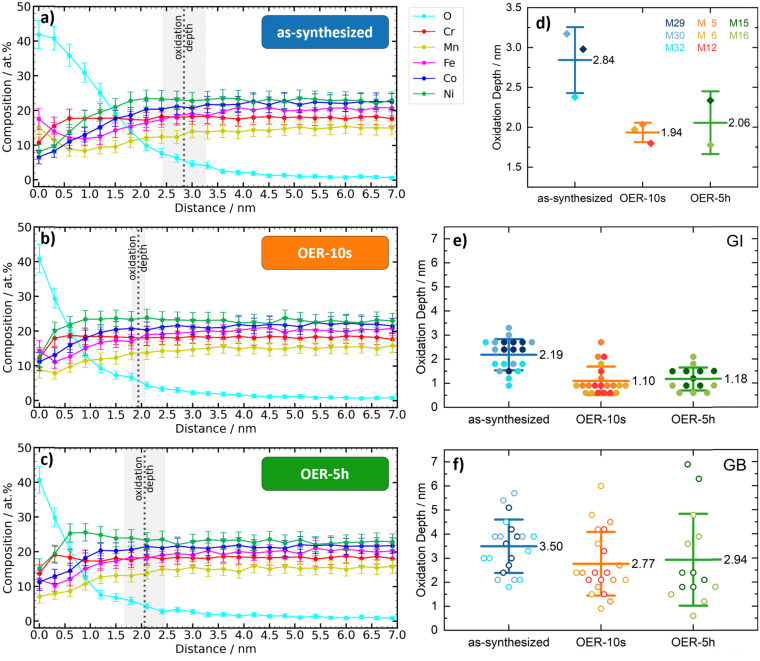
Oxidation depths. (a)–(c) Element composition *vs.* depth plots of the different states. Dashed lines indicate the mean oxidation depth with a standard deviation beyond which the oxygen content remains at low values. (d) Plot of the oxidation depths of the different states with mean value and standard deviation. Each tip is represented by a different color. (e) Plot of the oxidation depths of the different GI ROIs. (f) Plot of the oxidation depths of the different GB ROIs.

An overview about the oxidation depths of the different ROIs and states derived from APT is given in ESI,† Fig. S10. Interestingly, the oxygen-influenced surface layer thickness decreases after the OER potential is applied and remains stable even after 5 h of OER. The oxygen-influenced layer thickness ranges decrease from 2.84 ± 0.41 nm for the as-synthesized state to 1.94 ± 0.12 nm for the OER-10s state to 2.06 ± 0.39 nm for the OER-5h state (Fig. 3(a)–(d)). In this analysis, all ROIs were combined, and no distinction was made between GI and GB. The analysis of only GI or GB, shown in Fig. 3(e) and (f), respectively, shows that the trend is the same: the oxidization depth after application of the OER potential is lower than for the as-synthesized state, while the difference between the OER-10s and OER-5h states is quite small and the depth of the OER-5h state is slightly higher than for the OER-10s state. The comparison of the mean values between GI and GB ROIs shows that the oxidation depths are significantly higher in the GB regions: +60% for the as-synthesized state and about +150% for the states after OER (OER-10s: +152%, OER-5h: +149%). This result implies that oxidation under ambient conditions leads to a thicker oxygen-influenced surface layer than the harsh oxidation conditions of OER. It is important to note that already 10 s of OER is sufficient to change the depth-dependent oxygen amount, and even after 5 h of OER exposure, the oxidation depth remains the same. These results indicate that after OER the catalyst surface forms a stable surface oxide equilibrium with a thinner oxygen-influenced surface layer compared to the native oxygen-influenced surface layer of the as-synthesized state. This matches previous works, which show that the OER potential application leads to the formation of surface oxides in a HEA.^40,41^ These oxides were found to form a high entropy oxide (HEO) spinel phase and act as an active phase in the OER. A decent OER activity was also found for Cantor alloy HEO thin films, which were synthesized by reactive co-sputtering.^27^

In our EC-CPP approach we focus on the depth of the oxygen-influenced layer which comprises not only oxides but could also comprise oxide-related phases such as oxyhydroxides. To investigate the potential presence of oxyhydroxide(s) we further analyzed the APT data with focus on (i) H contents, and (ii) voltage evolution during APT data acquisition. Regarding point (i), although H ions are always produced in APT from the chamber and/or mainly from H contamination of the sample surface,^42^ a potential formation of hydroxide(s) should lead to a higher H content compared to the cases where no hydroxide forms. Based on this assumption, H contents in all states were quantified. However, no monotonic relation between H contents and different states could be established. Regarding point (ii), the evaporation field of native oxide may be different from that of the hydroxide(s). On this basis, the voltage evolution with ion sequence number for oxide is expected to be different from that of hydroxide. Fig. S11–S13 (ESI†) display the voltage evolution curves for the states as-synthesized, OER-10s, and OER-5h, respectively. All voltage evolution curves for the as-synthesized state exhibit a smooth logarithmic increase with ion sequence number. In contrast, the curves of the two EC-treated states show a steep linear increase at the low regime of ion sequence number, followed by a plateau. The shape of a voltage evolution curve is also determined by the geometry of an APT sample, including its tip radius and shank angle. Generally, a larger tip radius corresponds to a higher starting voltage for field evaporation, and a higher shank angle yields a higher slope of a voltage curve. In the current case, the tips of all three states are from the same microtip array and have similar geometry regarding the tip radius and shank angle. Therefore, the different shapes of the voltage evolution curves in Fig. S11–S13 (ESI†) should not result from the tip geometry, thus, the formation of hydroxide(s) during OER cannot be excluded.

### Change of the metal contents in the oxygen-influenced surface layer

**Fig. 4:**
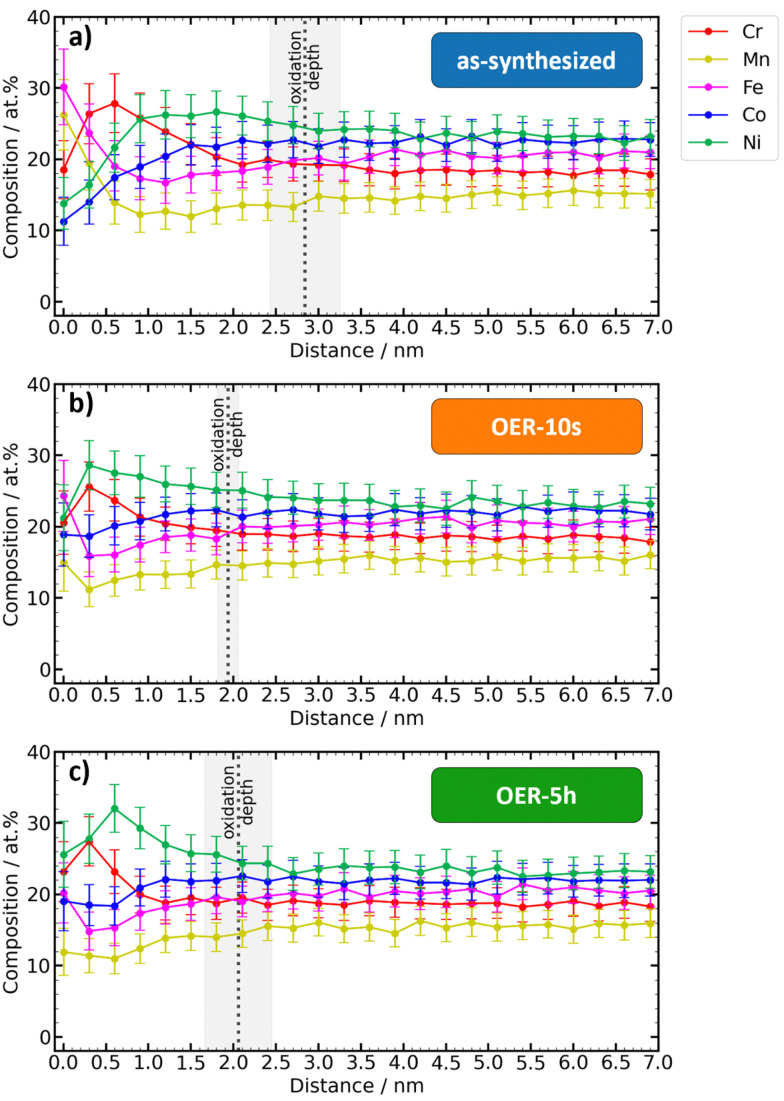
Depth-profiles of metal contents, normalized without oxygen. (a)–(c), Element composition *vs.* depth plots of the different states. Dashed lines indicate the mean oxidation depth with standard deviation beyond which the oxygen content remains at low values.

The metal content of the oxidized surface layer and the remaining metal volume below can change as a result of surface oxidation. For the investigation of the metal composition in the oxygen-influenced surface layer, the data is normalized without oxygen. The plot of the depth-profiles (Fig. 4) shows regions dominated by different oxides.

The metal contents in the very surface (0 nm) change from Fe > Mn > Cr > Ni > Co for the as-synthesized state to Fe > Ni > Cr > Co > Mn for the OER-10s state to Ni > Cr > Fe > Co > Mn for the OER-5h state. When comparing the order of element contents for the three states in the subsurface region (0.6 nm), the trend changes from Cr > Ni > Fe > Co > Mn for the as-synthesized state to Ni > Cr > Co > Fe > Mn for the OER-10s and OER-5h states. While the contents of Co, Mn, and Fe at the depth of 0.6 nm remain approximately at the same level for the as-synthesized and OER-5h state, the content of Ni steeply increases from 22 at% to 32 at% and the content of Co decreases from 28 at% to 23 at%, respectively. For all three states, the order of element contents in the volume of the film (3.6 nm) is approximately the same, *i.e.* Ni > Co > Fe > Cr > Mn. This is different from the passivity behavior of Cantor alloy under acidic non-electrochemical conditions, where besides Cr oxide, Co oxide, and Fe oxide, also Mn oxide can be found, but no Ni oxide.^34^

**Fig. 5:**
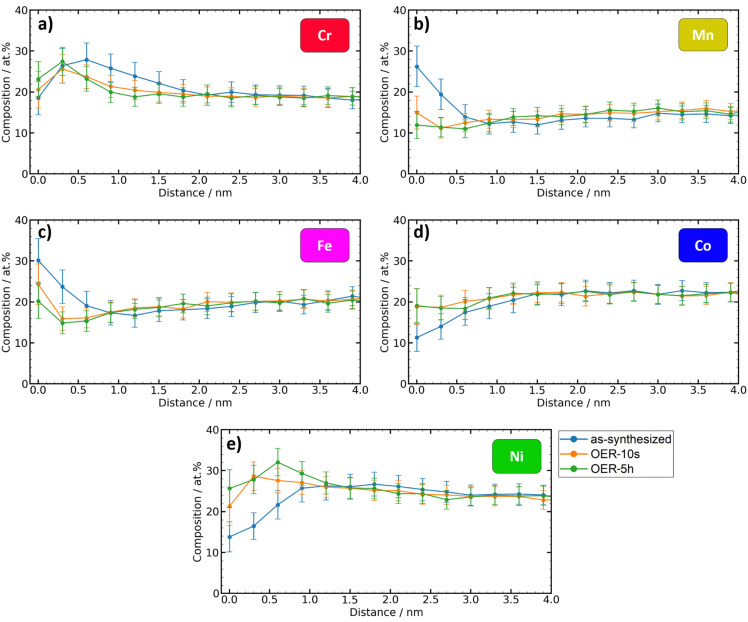
Comparison between the depth-dependent metal contents of the different states. (a)–(e) Element composition *vs.* depth plots of the metals. Metals are normalized without considering oxygen.

The elementwise plots for each state (Fig. 5) show that the biggest changes in the metal content occur in the first 2 nm of the top surface. Since electrochemical reactions occur at the catalyst surface, this finding has important implications for the understanding of composition–activity interpretations since the composition of the volume of the material, which is the quantity that is usually determined in synthesis and processing, can be significantly different from the relevant surface composition.

At the very top surface (0 nm), the as-synthesized state shows the highest contents of Mn and Fe as the most oxophilic constituents. Upon exposure to OER conditions, the top surface Mn content of 26 at% sharply decreases to 15 at% (OER-10s) and 12 at% (OER-5h). A similar trend is observed for Fe, that decreases from 30 at% to 24 at% (OER-10s) and 20 at% (OER-5h). On the other hand, the top surface content of Co and especially Ni increases under the OER conditions: from 11 at% to 19 at% (OER-10s and OER-5h) for Co and from 14 at% to 21 at% (OER-10s) and 26 at% (OER-5h) for Ni. The depth-profile of Cr content is the least affected by the exposure to the OER and the top surface content remains about the same (18–23 at%). Fig. 5(a) reveals that Cr shows very similar depth-profiles for the as-synthesized, OER-10s, and OER-5h state. A similar trend can be observed in the XPS analysis (Table S2, ESI†).

The difference between the curves of OER-10s and OER-5h is very small, which means that 10 s of applied OER potential was already enough to achieve a significant change in the surface composition of the catalyst. Further exposure to OER conditions only has a lower impact (only for Ni, the maximum content is more shifted to the volume). This shows that the equilibrium conditions manifest already after a very short time.

On the other hand, the difference between the states after EC exposure and the as-synthesized state is significant. To exclude that the exposure of the Cantor alloy thin film to ambient air after the OER experiment has an effect on the surface oxides, the data of the OER-10s and OER-5h states after 2 years of aging was also analyzed for comparison (see ESI,† Fig. S14). The results from the aged samples show a higher similarity to the as-synthesized state than to the states immediately after OER exposure (OER-10s and OER-5h), indicating that aging definitely has an effect on the surface oxides. However, the short exposure to ambient air after the EC experiment and before the transfer to the APT is not long enough to have a significant impact on surface reoxidation, since the elemental composition of OER-10s and OER-5h is still very different from the as-synthesized state.

Fig. 5 clearly shows that the relative atomic contents of Fe and Mn within 0–0.6 nm and Cr within 0.6–1.5 nm of the top surface for the OER-10s and especially the OER-5h state are significantly lower than those for the as-synthesized state. As the percentage of these elements decreases, the amount of Co and Ni increases accordingly. This, together with the reduction of the oxide layer thickness (Fig. 3), indicates that Cr, Mn, and Fe could dissolve to a certain degree during the OER.

In contrast to the thermal oxidation studies from Li *et al.*,^10^ where Cr and Mn oxides dominate the surface, the results match the data from single-element Pourbaix diagrams. At a pH of 12 and a potential of 1.7 V *vs.* RHE, corrosion of Cr, Mn, and Fe and passivation of Ni and Co occurs.^43^ This helps to explain the decreasing content of Cr, Mn, and Fe and the resulting increasing content of Ni and Co as oxides and/or oxyhydroxides in the catalyst surface in dependence on the OER potential.

In the case of Cr, the maximum element content is shifted from the volume of the catalyst film to its surface, where its amount remains almost constant in comparison to the other states. This means there is a maximum or saturation of Cr in the surface before it eventually goes into solution as CrO_4_^−^ ions. Therefore, it seems plausible that when the OER potential is applied, from the very surface of the film small amounts of Cr, Mn, and Fe may dissolve into the electrolyte, followed by rapid passivation through the formation of Co and Ni oxides and/or oxyhydroxides. This scenario is supported by both the APT measurements (Fig. 5) and the quantitative XPS analysis (Table S2, ESI†). Conclusively, the already existing data and knowledge of Pourbaix diagrams of the single elements can be used to roughly estimate the (pH-dependent) surface-composition change effect trends of CCSS electrocatalysts. However, the situation in the polyelemental Cantor alloy is more complex due to the electronic effects of the neighboring atoms. The investigation of stability of high entropy alloys could be further improved by coupling our electrochemical CPP approach with online ICP-MS analysis.^44^

## Conclusions

We applied a novel characterization technique, electrochemical CPP, which enables high-throughput APT analysis with near atomic-scale resolution of surface compositional changes of catalyst materials due to electrochemical exposures. These changes, *i.e.*, differences between surface and volume compositions, can be quantified and followed over different synthesis and processing states, which is of high importance for designing polyelemental electrocatalysts. Using these unique capabilities in analyzing the first atomic layers for the exemplary case of a Cantor alloy film, allowed us to draw the following conclusions on the self-synthesis of surface oxides of a CCSS. A very thin surface oxide layer is established by the exposure to OER, even thinner than the native oxide. This happens fast, already 10 s are enough to create an oxide passivation layer in the surface that is not affected by longer applied potential. This is highly beneficial for electrocatalysts as the very thin oxide is intimately coupled to the conducting sub-surface. The interaction of the surface metals with the electrolyte under OER conditions leads to a specific change of the surface metal ratios, which is different to native or thermal oxides of the Cantor alloy. This change can be rationalized using the Pourbaix diagrams of the different elements. For single elements at a pH of 12 and an applied potential of 1.7 V *vs.* RHE, Cr, Mn, and Fe corrode, and Co and Ni contribute as passivated oxides and potentially oxyhydroxides to the oxygen-influenced surface layer. The observed behavior is an additional favorable effect of compostionally complex alloys (high entropy alloys) for electrocatalysis. We found that the electrochemical exposure effect on composition of the compositionally complex surface dominates over thermal or oxidation exposure. The overall findings can be used in future work to direct the evolution of surface oxides on high entropy alloys to obtain sustainable electrocatalysts with high activity and stability.

## Author contributions

A. L. and W. S. supervised and coordinated the project. A. L. and T. L. designed the project. V. S. synthesized the EC-CPP. V. S. and T. L. performed the electrochemical measurements. Y. L. performed the APT experiments and analyzed the APT data. V. S. interpreted the APT data and did further data analyses and data plots. A. K. performed the TEM measurement and analysis. F. L. performed the XPS measurements and analysis. All authors agreed on the contents and conclusion of the paper.

## Data availability

The data supporting this article have been included as part of the ESI.†

## Conflicts of interest

The authors declare no competing financial interests.

## Supplementary Material

MH-011-D4MH00245H-s001
