# Supplementary material for: Self-formation of compositionally complex surface oxides on high entropy alloys observed by accelerated atom probe tomography: a route to sustainable catalysts
Source: Mater Horiz. 2024 Jul 18;11(20):4932–41. doi: 10.1039/d4mh00245h (PMC11472866; doi:10.1039/d4mh00245h)
Supplement: MH-011-D4MH00245H-s001 [file MH-011-D4MH00245H-s001.pdf]

Supplementary Information (SI) for Materials Horizons.
This journal is © The Royal Society of Chemistry 2024

Supporting Information

Methods, extended results including data overview, representative in detail tip analysis of the different states and oxidation depth of the different regions of interest (ROIs).

Methods

Synthesis of EC-CPPs. The CPP were fabricated by sputter deposition in a magnetron sputter system (AJA POLARIS-5, AJA International) with a DC power supply (DC-XS 1500 from AJA International Inc., North Scituate) and a 1.5-inch diameter $Cr_{18}Mn_{20}Fe_{20}Co_{21}Ni_{21}$ alloy target with a purity of 99.95%. It was sputtered with rotation of the substrate holder on a commercially available tip array (Microtip™ arrays, CAMECA Instruments, Inc.) of 36 pre-sharpened Si tips at a pressure of 0.5 Pa and a power of 30 W over a time of 6480 s to achieve a film thickness of 100 nm. The CPPs were subsequently annealed in vacuum for 2.5 h at 100°C, 1 h at 150°C, and 1 h at 200°C to ensure a better tip stability in APT. The resulting state is called as-synthesized state.

Electrochemical Experiments. Electrochemical measurements were performed with a pgu-BI 100 potentiostat in a three-electrode setup using a Ag/AgCl (3 M KCl) reference electrode and a Pt wire counter electrode, located in a separate compartment. The EC-CPP with the Cantor alloy thin film was used as the working electrode. KOH (0.1 M) was used as electrolyte and the applied potential was 1.7 V vs RHE. The potential of 1.7 V was chosen to be clearly in the OER range but simultaneously avoid excessive bubble formation by recording a linear sweep voltammogram (LSV) (Fig. S1). The equation for the conversion of the potential from the Ag/AgCl (3 M KCl) to RHE scale is given in Eq. S1 with E_0(Ag/AgCl/3 M KCl) = 0.207 V.

Nernst equation for the calculation of RHE potential at 25°C

Eq. S1

$$E(RHE) = E_0(Ag/AgCl/3M\ KCl) + 0.059 \cdot pH + E(Ag/AgCl)$$

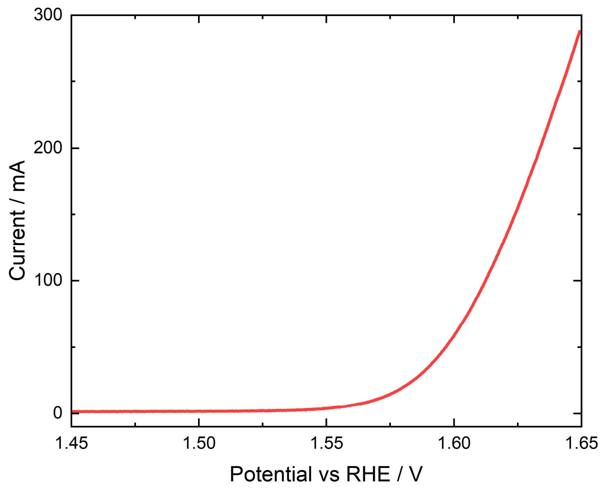

Fig. S1 | LSV of an EC-CPP in 0.1 M KOH, coated with Cantor alloy.

APT measurements. APT measurements were performed on a LEAP 5000 XR™ (CAMECA Instruments) using voltage pulsing at 70 K with 20% pulse fraction at a pulse repetition rate of 200 kHz and a detection rate of 0.004 atoms per pulse. The APT data were reconstructed and analyzed by IVAS 3.8.10 software.

Two measures were taken to minimize possible loss of surface atoms. First, a voltage-pulsing mode was applied instead of a laser-pulsing mode to eliminate the loss of surface atoms caused by the latter during laser alignment; second, since there were 36 identical tips available on each CPP, several tips were used as test samples to find the ideal position of a tip and to determine the starting voltage under which surface ions evaporate at the target detection rate. By doing so, a new APT tip can be directly measured, and the data of surface composition with high signal-to-noise ratio can be derived.

TEM measurements. Samples for TEM measurements were prepared using a FEI Helios G4 CX focused ion beam (FIB) system operated at 30 kV. In the first step, an electron beam carbon deposition was applied to protect the sample surface from Ga-ions. The CPP sample lift-out and transfer to a TEM grid were performed using redeposition. FEI Tecnai F20 G2 S-Twin TEM operated at 200kV was used to characterize the sample after deposition of the CPPs'. After OER the TEM was carried out on an aberration, probe-corrected JEOL JEM-ARM200F (NEOARM) operated at 200 kV.

XPS measurements. XPS measurements were performed on an additional Cantor alloy thin film which was deposited on a flat 7 x 7 mm^2 Si substrate and was measured before and after exposure to OER-5h. The synthesis process including deposition and annealing was equal to those of the films fabricated on the EC-CPP chips (see section "Synthesis of EC-CPPs"). The wetted area during the OER exposure was 5 x 7 mm^2. The XPS measurements were carried out on a Kratos Axis Nova, using a monochromatic Al Kα X-ray source operated at 180 W (15 mA, 12 kV), and a delay-line detector with pass energies of 20 eV (high-resolution scans) and 160 eV (survey scans). The electron emission angle was 0°, the charge neutralizer was turned on, and there was no sputter etching applied. The pressure during the measurements was below 1.8 x 10^{-6} Pa. Survey scans and high-resolution scans of 300 x 700 μm^2 analysis areas for the Cr 2p, Mn 2p, Fe 2p, Co 2p, Ni 2p, O 1s and C 1s regions were measured. The latter was only used for charge correction (adventitious C-C at 284.8 eV). Quantification was done using Shirley backgrounds and the Kratos ESCApe software with its pre-defined relative sensitivity factors.

Overview about the data

For each state, the data from at least two different tips was used (Tab. S1). The same number of ROIs was taken equally distributed from the grain interior (GI) and the grain boundaries (GB).

Tab. S1 | Overview about the different tips for each state with the number of ROIs from GI and GB.

**State**	**APT tip**	**Number of ROIs**	
		**GI**	**GB**
as-synthesized	M29	7	7
	M30	7	7
	M32	7	7
OER-10s	M5	7	7
	M6	7	7
	M12	7	7
OER-5h	M15	7	7
	M16	7	7

Analysis of representative tips for each state

Figs. S2, S3, and S5 show the analysis of one tip for each state representatively. 14 ROIs, randomly distributed, were selected from the GI and the GB equally (a and b). For further analysis, the composition vs depth plots of the 14 ROIs were summed up and normalized to one plot (c). For showing the differences in the oxidation depth between GI and GB, these summed up plots were plotted in d and e. The oxidation depth for GB is deeper than for GI, but the general trend between the different states remains the same.

All 14 ROIs from the representative tips are plotted in Fig. S4, S6, and S7, respectively.

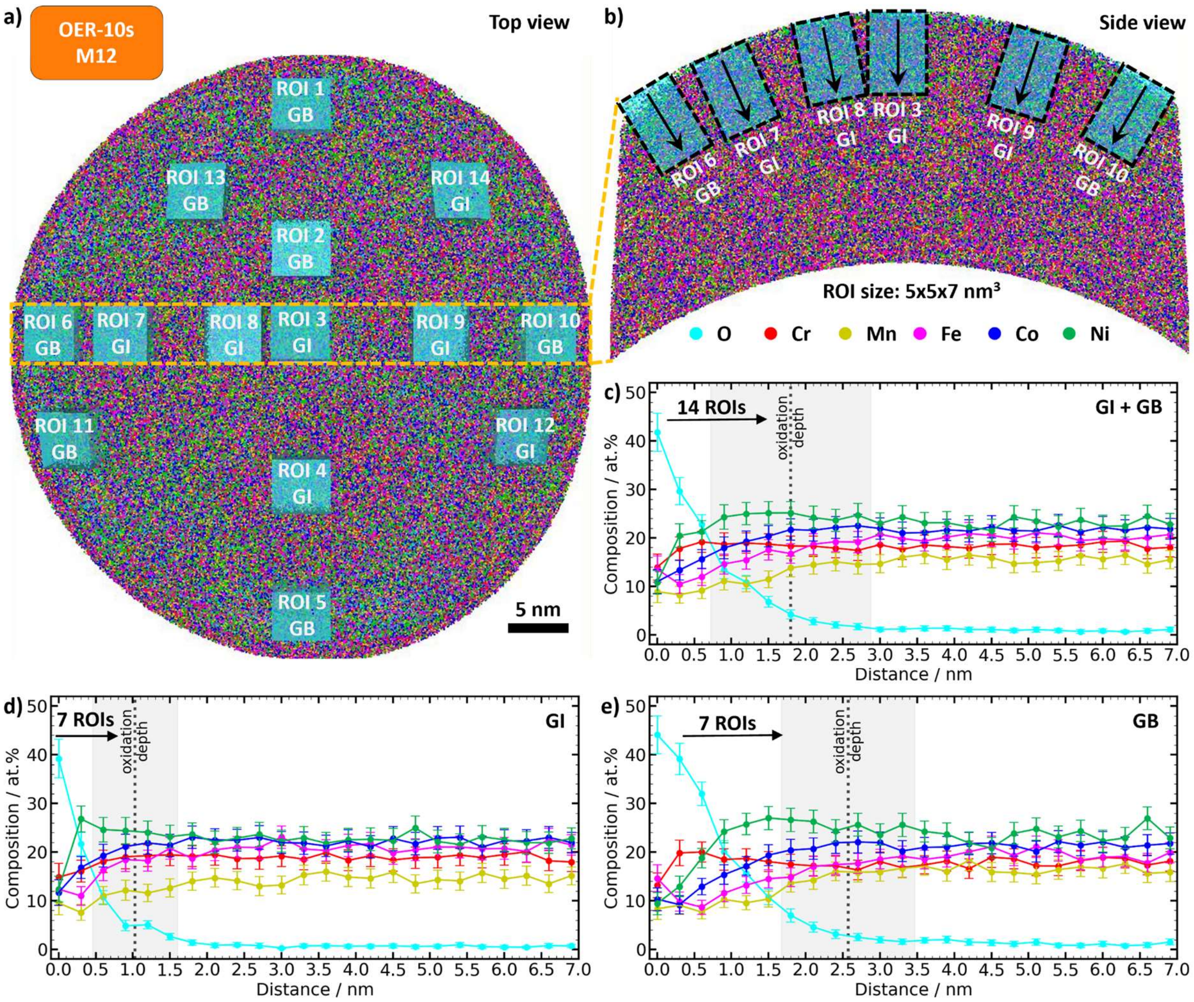

Fig. S2 | Results of the APT analysis of the OER-10s state, measured on a representative tip from the EC-CPP. a, Top view of the tip M12 and **b** side view of the 5-nm thin slice marked in yellow in the top view. The ROIs are marked by blue rectangles. The arrows show the direction of the composition profiles. **c**, **d**, and **e** show depth-dependent composition plot of merged and averaged ROIs of the tip with marked mean oxidation depth with standard deviation. The error of the APT measurements is indicated. **c** includes all 14 ROIs of GI and GB, **d** includes the 7 ROIs of GI and **e** includes the 7 ROIs of GB.

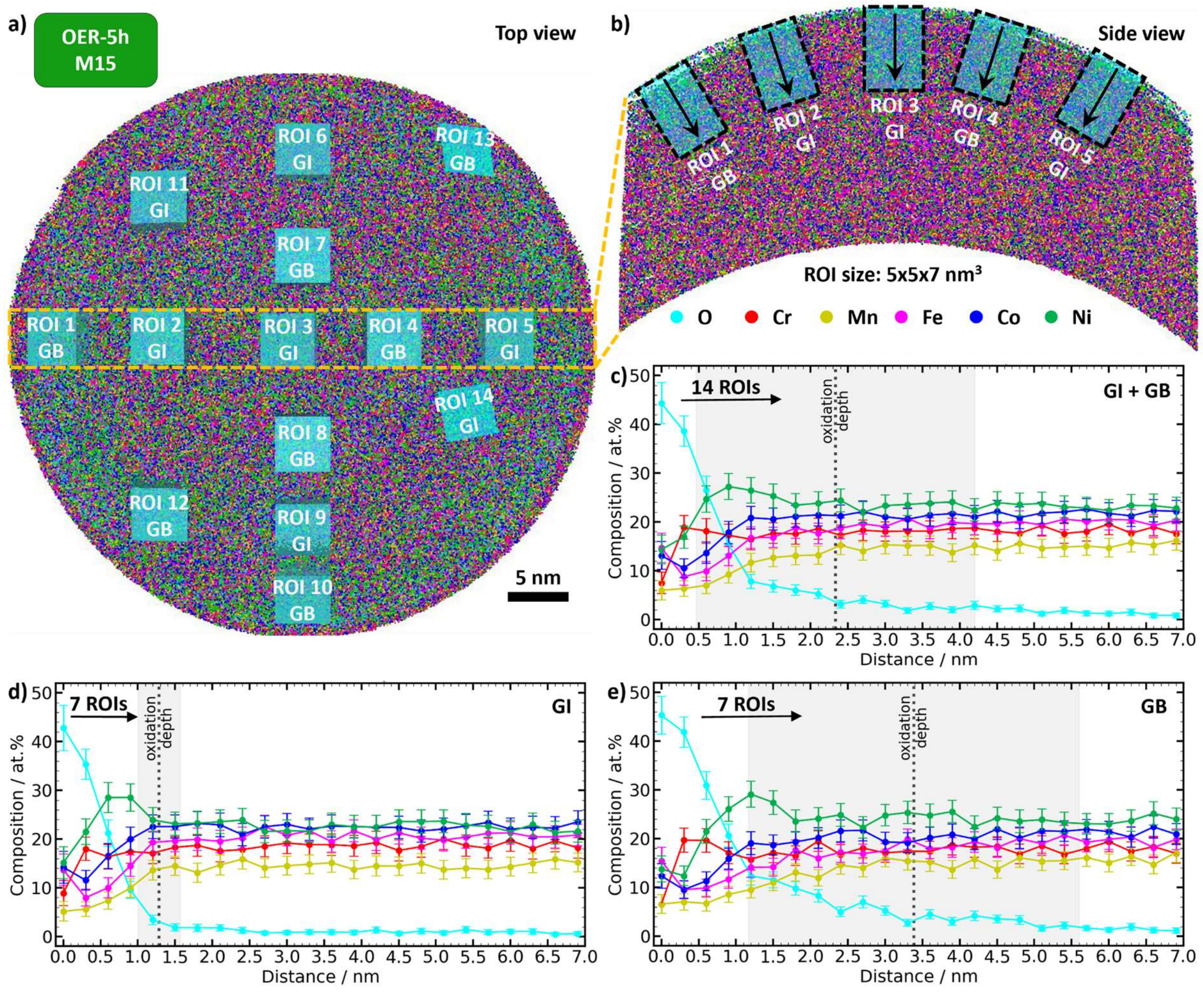

Fig. S3 | Results of the APT analysis of the OER-5h state, measured on a representative tip from the EC-CPP. a, Top view of the tip M15 and **b** side view of the 5-nm thin slice marked in yellow in the top view. The ROIs are marked by blue rectangles. The arrows show the direction of the composition profiles. **c**, **d**, and **e** show depth-dependent composition plot of merged and averaged ROIs of the tip with marked mean oxidation depth with standard deviation. The error of the APT measurements is indicated. **c** includes all 14 ROIs of GI and GB, **d** includes the 7 ROIs of GI and **e** includes the 7 ROIs of GB.

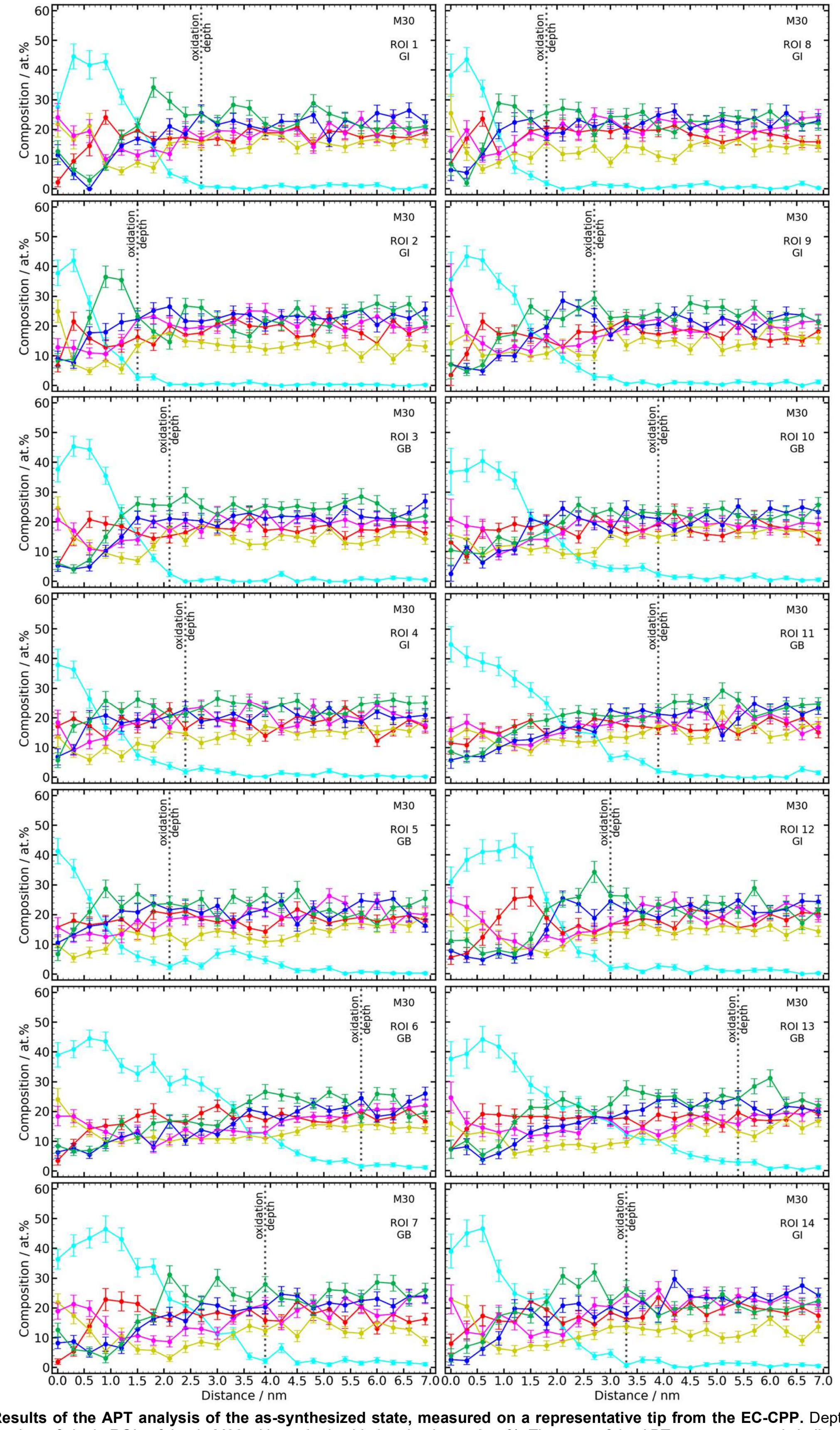

Fig. S4 | Results of the APT analysis of the as-synthesized state, measured on a representative tip from the EC-CPP. Depth-dependent composition plots of single ROIs of the tip M30 with marked oxidation depth at < 3 at.%. The error of the APT measurements is indicated.

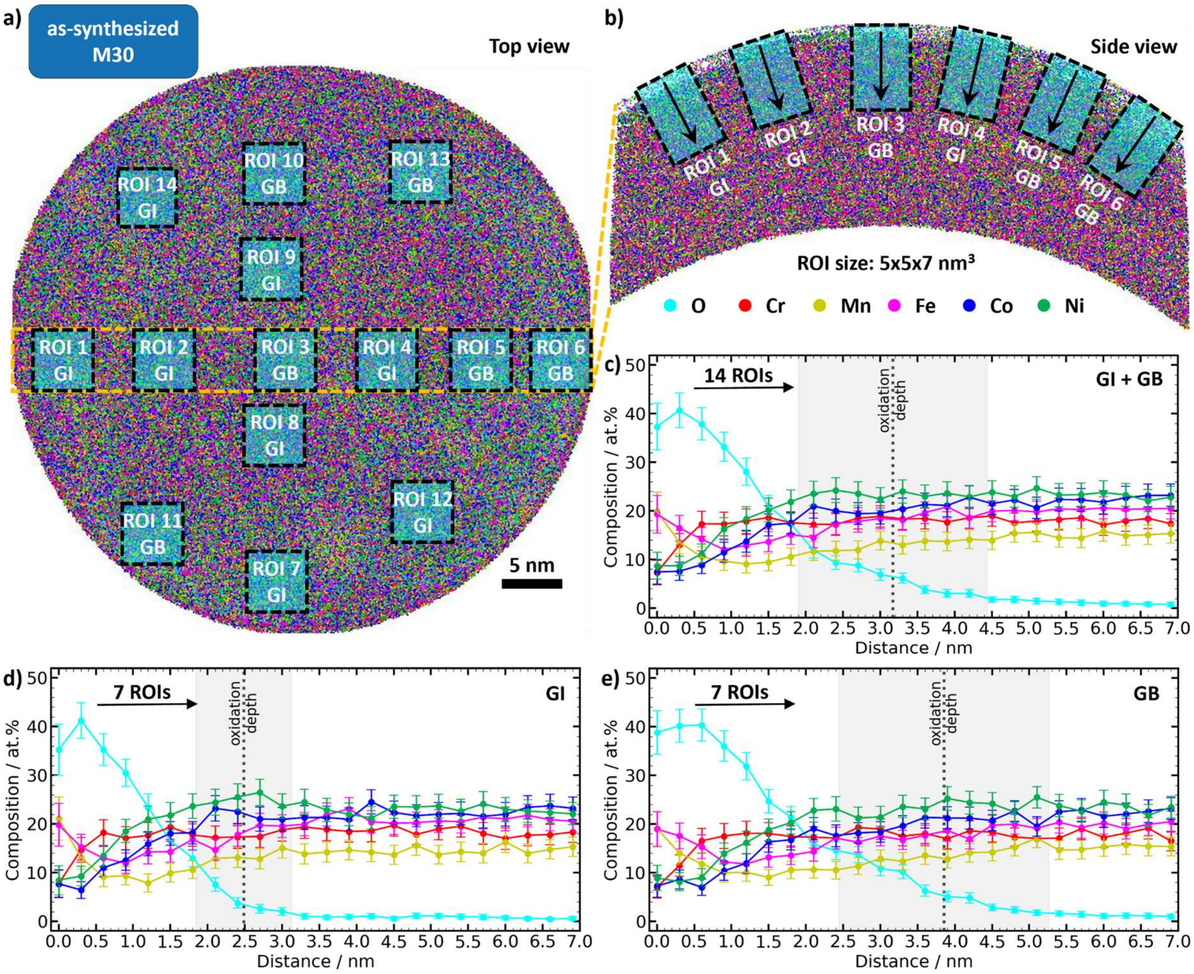

Fig. S5 | Results of the APT analysis of the as-synthesized state, measured on a representative tip from the EC-CPP. **a**, Top view of the tip M30 and **b** side view of the 5-nm thin slice marked in yellow in the top view. The ROIs are marked by blue rectangles. The arrows show the direction of the composition profiles. **c**, **d**, and **e** show depth-dependent composition plot of merged and averaged ROIs of the tip with marked mean oxidation depth with standard de-viation. The error of the APT measurements is indicated. **c** includes all 14 ROIs of GI and GB, **d** includes the 7 ROIs of GI and **e** includes the 7 ROIs of GB.

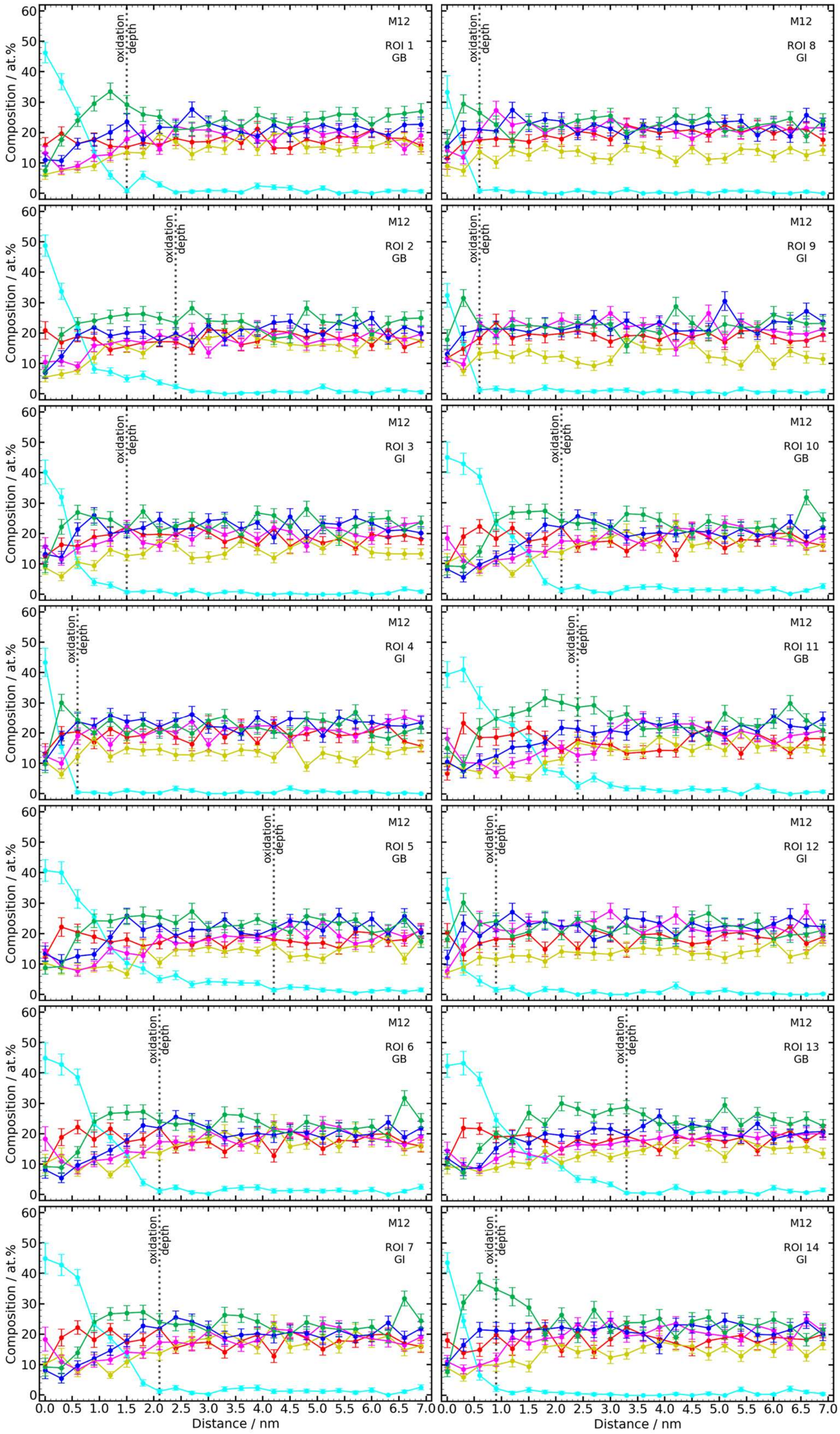

Fig. S6 | Results of the APT analysis of the OER-10s state, measured on a representative tip from the EC-CPP. Depth-dependent composition plots of single ROIs of the tip M12 with marked oxidation depth at < 3 at.%. The error of the APT measurements is indicated.

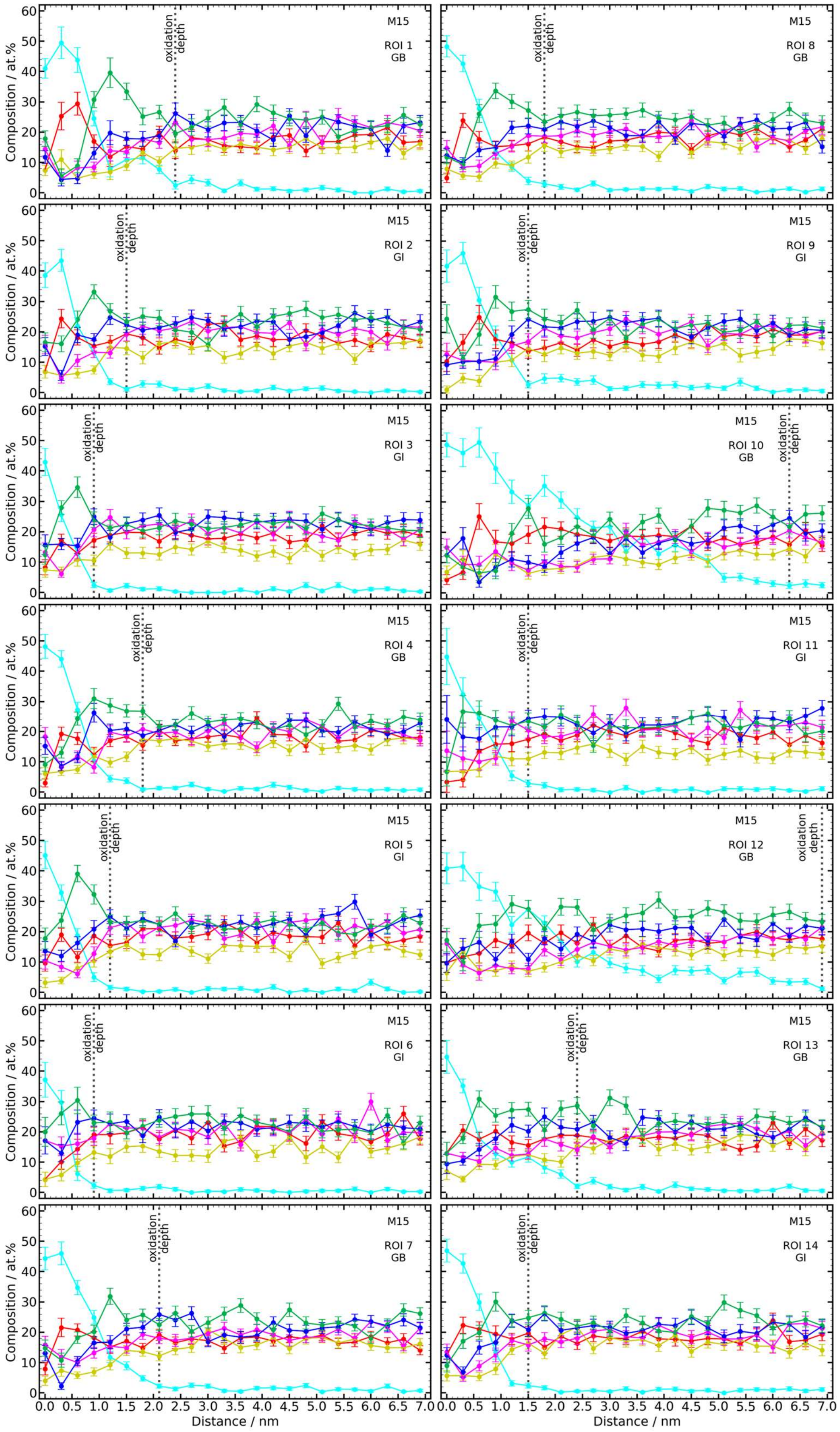

Fig. S7 | Results of the APT analysis of the OER-5h state, measured on a representative tip from the EC-CPP. Depth-dependent composition plots of single ROIs of the tip M15 with marked oxidation depth at < 3 at.%. The error of the APT measurements is indicated.

XPS analysis

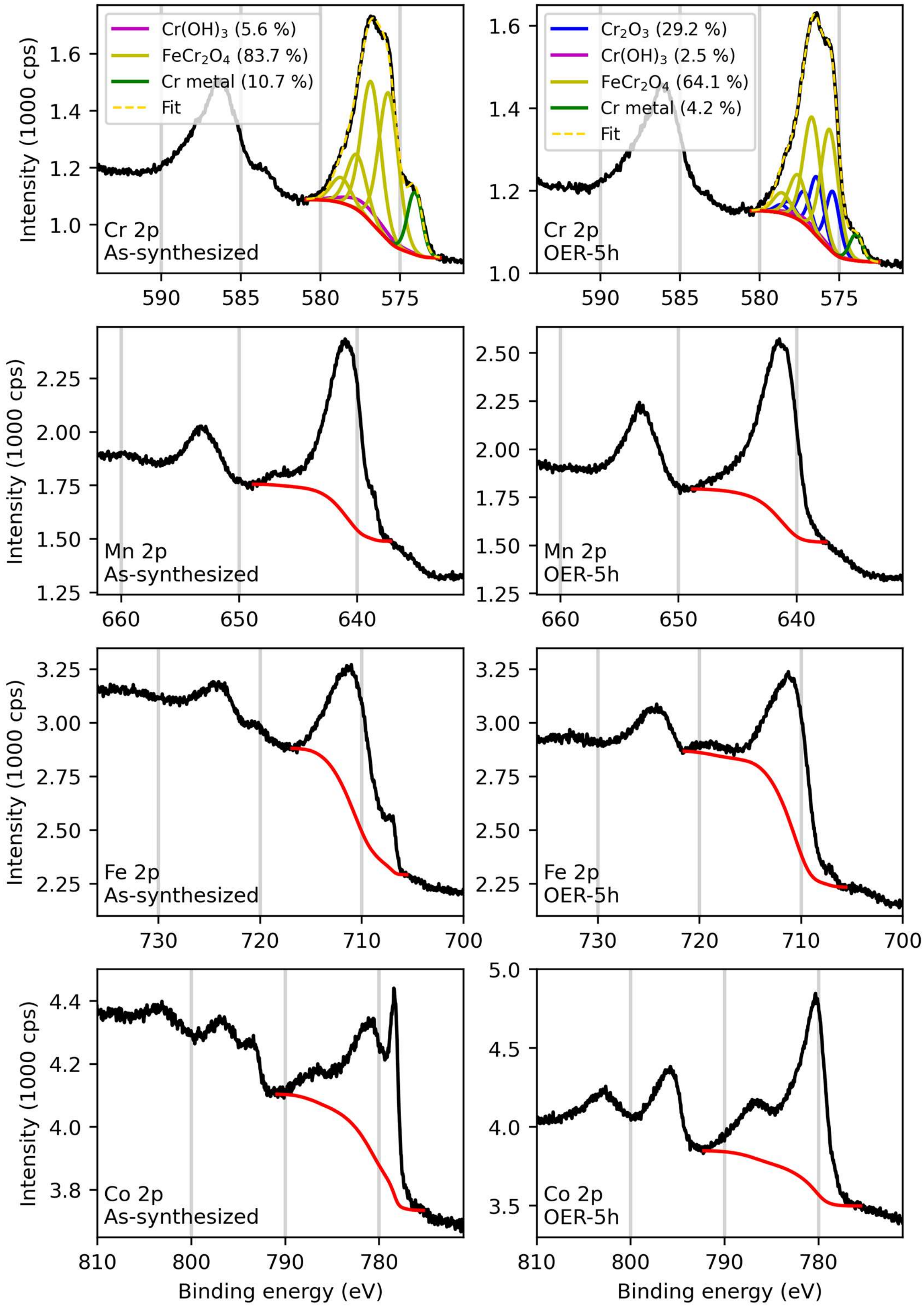

Fig. S8 | XPS characterization of a Cantor alloy thin film before and after OER-5h. Part 1: High-resolution scans of Cr 2p, Mn 2p, Fe 2p, and Co 2p. Black: Measured XPS spectra, red: Shirley backgrounds used for quantification.

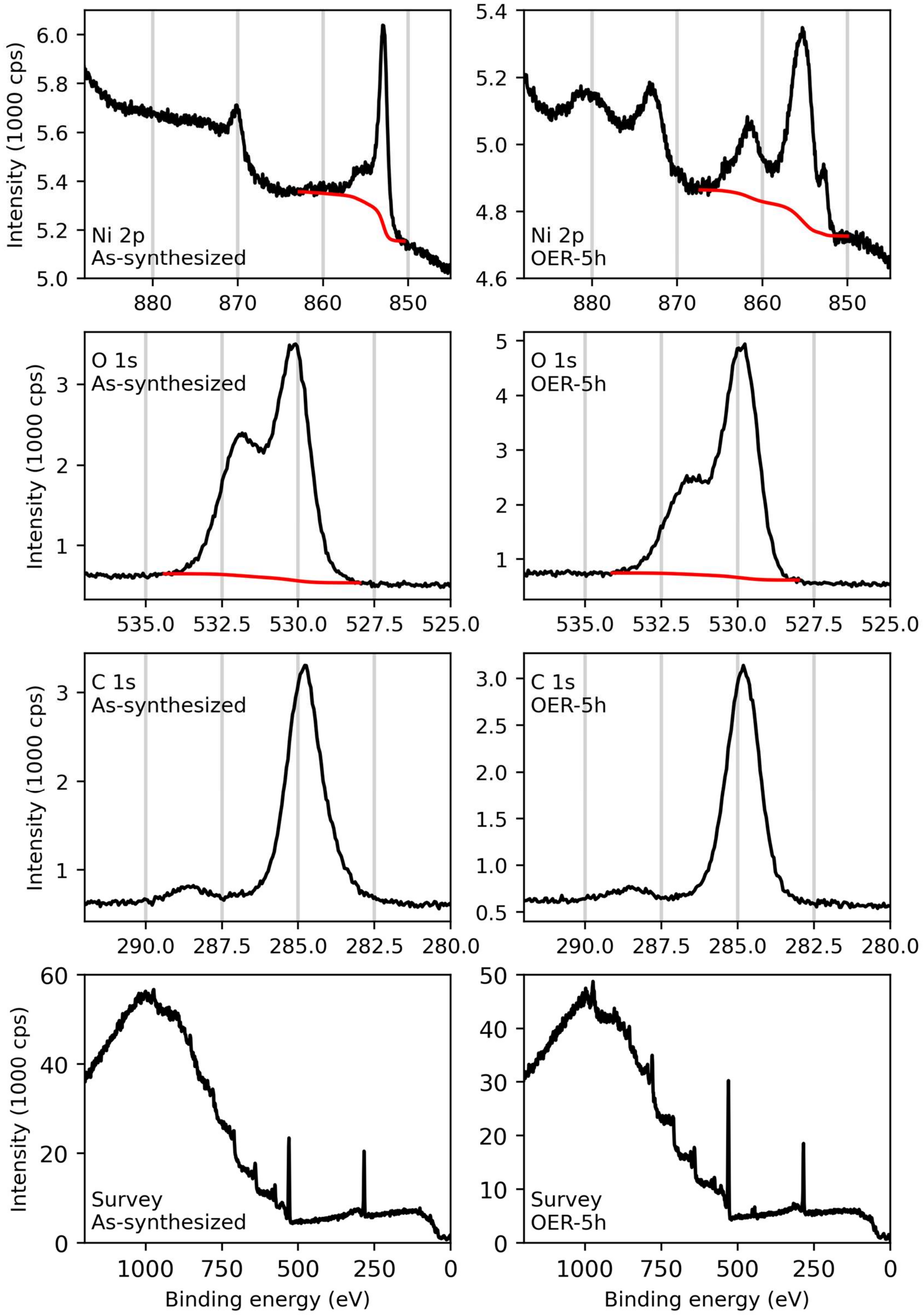

Fig. S9 | XPS characterization of a Cantor alloy thin film before and after OER-5h. Part 2: High-resolution scans of Ni 2p, O 1s, C 1s, and survey scans. Black: Measured XPS spectra, red: Shirley backgrounds used for quantification.

Cr: The Cr 2p region is the only one that does not overlap with any potential Auger peak and therefore, detailed peak fitting was performed by using the exact peak fitting parameters from Biesinger et al.[1]

Mn: Mn 2p spectra in the as-synthesized state and after the OER are very similar. As-syn.: Small shoulder of metallic Mn (appr. 638.7 eV) and main peak of Mn-oxides/-hydroxides (appr. 641 eV). OER-5h: The shoulder of metallic Mn disappeared. Overlap with Ni LMMa Auger peak (appr. 640.5 eV) prohibits conclusive peak fitting.

Fe: As-syn.: Main peak (appr. 711 eV) indicates Fe-oxide/-hydroxide, small shoulder (appr. 707.1 eV) originates from metallic Fe. OER-5h: Hardly any metallic Fe. Overlap with Co LMM Auger (appr. 716 eV) and Ni LMMb (706.7 eV) prohibits conclusive peak fitting.

Co: As-syn.: Sharp peak at appr. 778.4 eV is metallic Co, peak at appr. 781 eV originates from Co-oxide/-hydroxides. OER-5h: No visible component of metallic Co, instead more intense Co-oxide/-hydroxide peak. Overlap with Fe LMMa Auger peak (appr. 786 eV) prohibits conclusive peak fitting.

Ni: As-syn.: The sharp peak at appr. 853 eV is from metallic Ni. The shoulder at appr. 855 eV could either be Mn LMMc Auger peak or Ni-oxide/hydroxide. OER-5h: A small visible peak of metallic Ni remained (853 eV), and two new peaks at appr. 855.4 eV and 861.6 eV appeared. These two new peaks look qualitatively very similar to the spectra of $NiFe_2O_4$ from Biesinger et al.[1] However, in our case, there is an overlap with a potential Mn LMMc Auger peak at. Appr. 852.7 eV, which is why the fitting model from Biesinger et al. does not lead to a good fit of our measured spectrum (OER-5h).

O: As there are so many possible oxides with Cr, Mn, Fe, Co and Ni, peak fitting of the O 1s spectra is very ambiguous. In general, the component at app. 530 eV can be ascribed to metal-oxides, and the component at app. 532 eV to organic species or adsorbed water. Metal-hydroxides would be expected at intermediate binding energies, appr. 530.5 – 531.8 eV.

C: C 1s spectrum does not change upon OER-5h. It remains a shape that is characteristic for adventitious C (i.e. organic species always found at any surface). The main peak (C-C species) was shifted to 284.8 eV and used for charge correction of all measured spectra.

Survey: Survey scans were used to show that no additional elements are present except Cr, Mn, Fe, Co, Ni, O and C.

Tab. S2 | Quantitative XPS analysis resulting from the spectra shown in Fig. S8 and S9. The results are subject to error because of several potential overlaps with Auger peaks.

**State**	**Cr (at. %)**	**Mn (at. %)**	**Fe (at. %)**	**Co (at. %)**	**Ni (at. %)**	**O (at. %)**
As-synthesized	10.0	11.2	10.8	7.5	3.3	57.2
OER-5h	5.1	10.8	8.5	13.1	5.4	57.1

[1] M. C. Biesinger, B. P. Payne, A. P. Grosvenor, L. W. Lau, A. R. Gerson and R. S. Smart, Resolving surface chemical states in XPS analysis of first row transition metals, oxides and hydroxides: Cr, Mn, Fe, Co and Ni, *Appl. Surf. Sci.*, 2011, **257**, 2717–2730.

Oxidation depths of the different ROIs

The oxidation depth for each ROI is plotted in Fig. S1, where the colors mark the different tips. GI and GB are distinguished by filled and empty symbols.

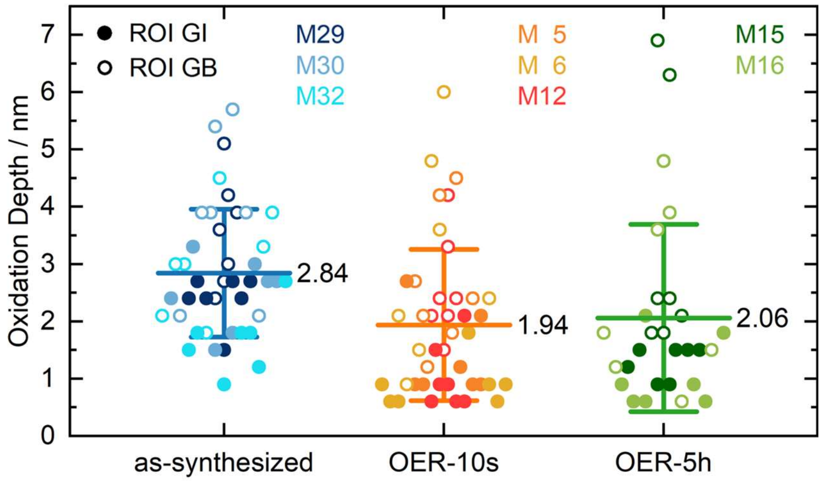

Fig. S10 | Oxidation depths of the different ROIs with mean and standard deviation. The colors represent different tips.

Examples of voltage evolution with ion sequence number during APT data acquisition

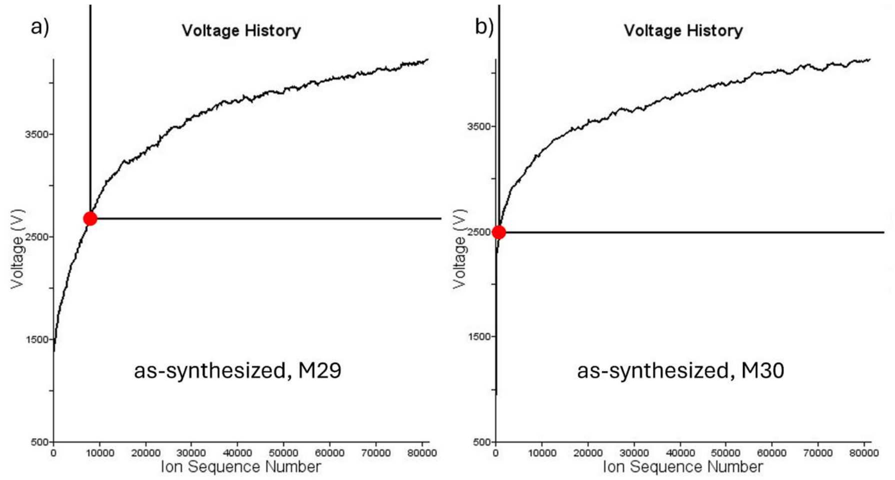

Fig. S11 | Examples of voltage evolution with ion sequence number during APT data acquisition for representative tips of the as-sythesized state. Red symbols mark the starting voltages for data analysis.

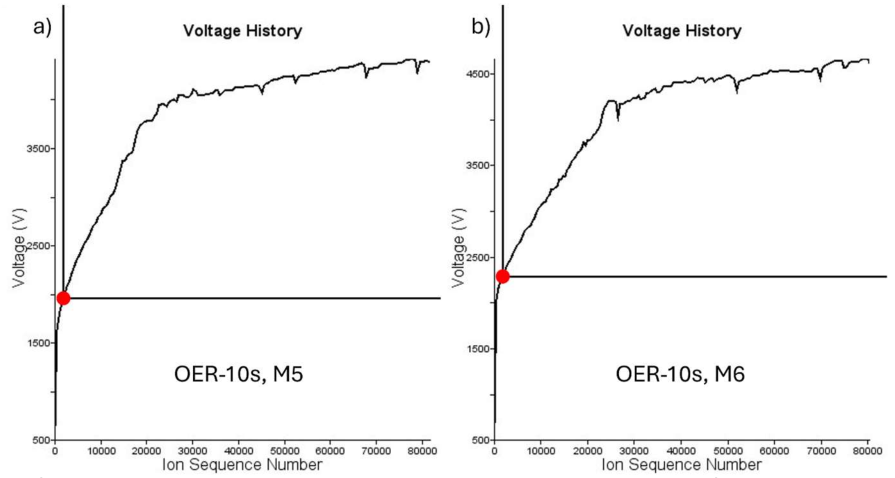

Fig. S12 | Examples of voltage evolution with ion sequence number during APT data acquisition for representative tips of the OER-10s state. Red symbols mark the starting voltages for data analysis.

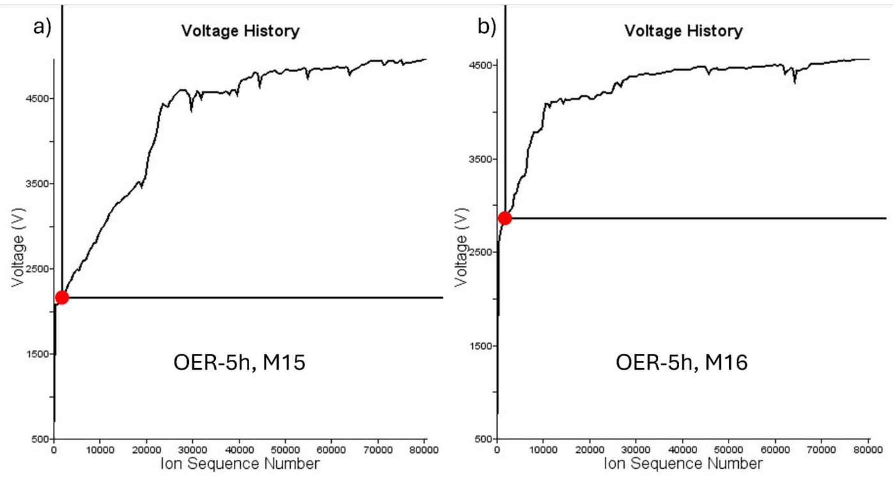

Fig. S13 | Examples of voltage evolution with ion sequence number during APT data acquisition for representative tips of the OER-5h state. Red symbols mark the starting voltages for data analysis.

Metal contents of OER-10s and OER-5h after two years of aging

Fig. S14 shows the elementwise plots of OER-10s and OER-5h after 26h at 200°C and subsequent 2 years in the desiccator. The data is processed and plotted as the original data in the main manuscript in Fig. 5, using 2 tips for aged OER-10s and 1 tip for aged OER-5h.

Compared to the original measurements, the very top surface (0 nm) of the aged OER-10s and the aged OER-5h contains more Mn (20 and 16 at%) and Fe (34 and 25 at%), less Cr (7 and 21 at%) and Ni (20 and 19 at%), and a similar amount of Co (18 and 19 at%). In the subsurface region (0.6 nm), the aged tips contain less Cr (21 and 16 at%) and Mn (8 and 9 at%), more Fe (19 and 20 at%) and Co (23 and 24 at%), and a similar amount of Ni (28 and 31 at%). The volume of the film (3.6 nm) shows no significant differences in the element contents between the original and the aged data.

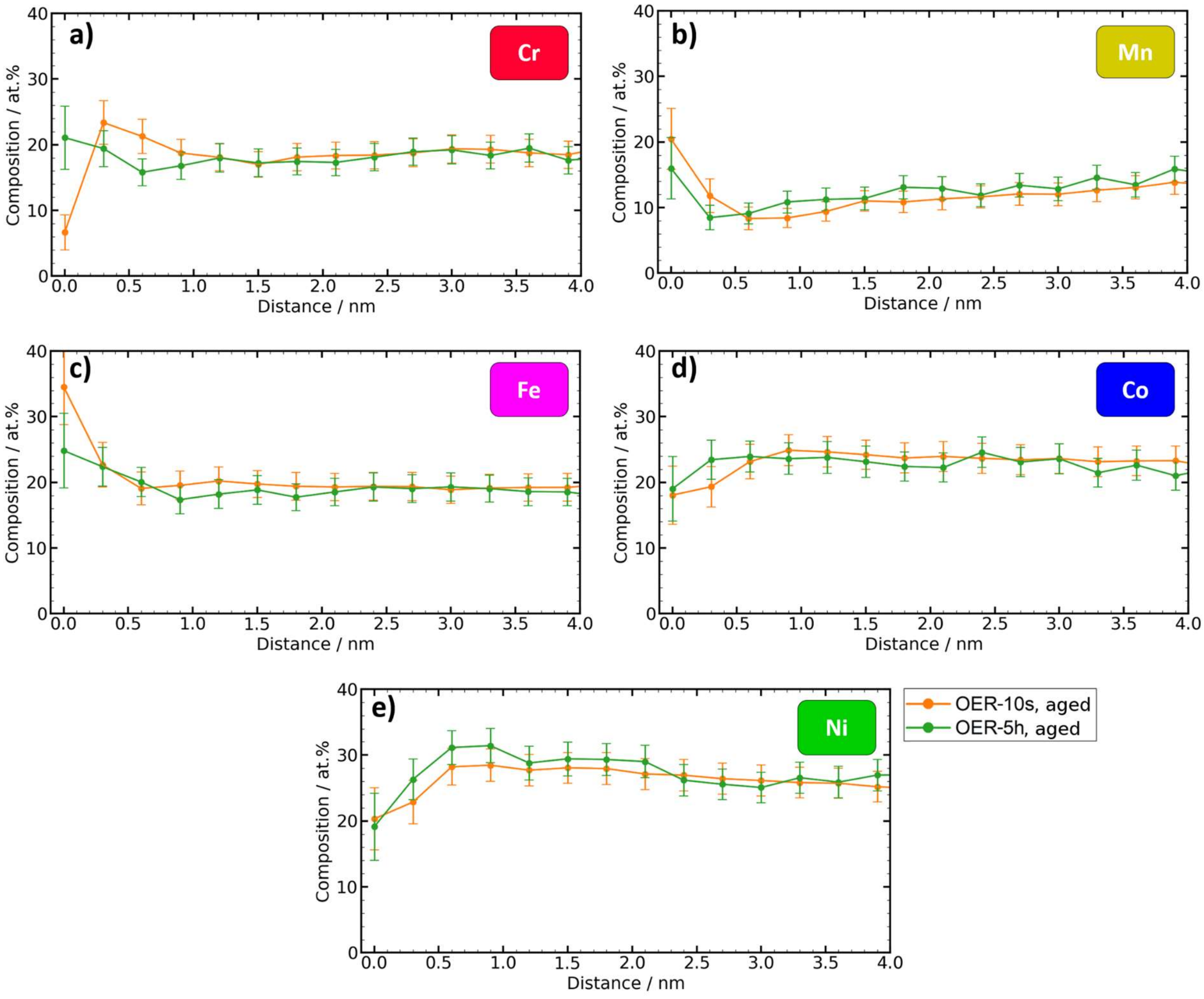

Fig. S14 | Comparison between the depth-dependent metal contents of the different aged states after OER exposure. a-e, Element composition vs depth plots of the metals. Metals are normalized without considering oxygen.
